# Transcriptome Analysis of Atlantic Salmon (*Salmo salar*) Skin in Response to Sea Lice and Infectious Salmon Anemia Virus Co-Infection Under Different Experimental Functional Diets

**DOI:** 10.3389/fimmu.2021.787033

**Published:** 2022-01-03

**Authors:** Wenlong Cai, Surendra Kumar, Umasuthan Navaneethaiyer, Albert Caballero-Solares, Laura A. Carvalho, Shona K. Whyte, Sara L. Purcell, Nellie Gagne, Tiago S. Hori, Melissa Allen, Richard G. Taylor, Rachel Balder, Christopher C. Parrish, Matthew L. Rise, Mark D. Fast

**Affiliations:** ^1^ Department of Pathology and Microbiology, Atlantic Veterinary College, University of Prince Edward Island, Charlottetown, PE, Canada; ^2^ Department of Infectious Diseases and Public Health, Jockey Club College of Veterinary Medicine and Life Sciences, City University of Hong Kong, Hong Kong, Hong Kong SAR, China; ^3^ Department of Ocean Sciences, Memorial University of Newfoundland, St. John’s, NL, Canada; ^4^ Fisheries and Oceans Canada, Moncton, NB, Canada; ^5^ Centre for Aquaculture Technologies Canada, Souris, PE, Canada; ^6^ Cargill Animal Nutrition, Elk River, MN, United States

**Keywords:** Atlantic salmon, sea lice, infectious salmon anemia virus (ISAv), co-infection, transcriptome, functional diets, immune response

## Abstract

Sea lice (*Lepeophtheirus salmonis*) are ectoparasitic copepods that cause significant economic loss in marine salmoniculture. In commercial salmon farms, infestation with sea lice can enhance susceptibility to other significant pathogens, such as the highly contagious infectious salmon anemia virus (ISAv). In this study, transcriptomic analysis was used to evaluate the impact of four experimental functional feeds (i.e. 0.3% EPA/DHA+high-ω6, 0.3% EPA/DHA+high-ω6+immunostimulant (IS), 1% EPA/DHA+high-ω6, and 1% EPA/DHA+high-ω3) on Atlantic salmon (*Salmo salar*) during a single infection with sea lice (*L. salmonis*) and a co-infection with sea lice and ISAv. The overall objectives were to compare the transcriptomic profiles of skin between lice infection alone with co-infection groups and assess differences in gene expression response among animals with different experimental diets. Atlantic salmon smolts were challenged with *L. salmonis* following a 28-day feeding trial. Fish were then challenged with ISAv at 18 days post-sea lice infection (dpi), and maintained on individual diets, to establish a co-infection model. Skin tissues sampled at 33 dpi were subjected to RNA-seq analysis. The co-infection’s overall survival rates were between 37%-50%, while no mortality was observed in the single infection with lice. With regard to the infection status, 756 and 1303 consensus differentially expressed genes (DEGs) among the four diets were identified in “lice infection vs. pre-infection” and “co-infection vs. pre-infection” groups, respectively, that were shared between the four experimental diets. The co-infection groups (co-infection vs. pre-infection) included up-regulated genes associated with glycolysis, the interferon pathway, complement cascade activity, and heat shock protein family, while the down-regulated genes were related to antigen presentation and processing, T-cell activation, collagen formation, and extracellular matrix. Pathway enrichment analysis conducted between infected groups (lice infection vs. co-infection) resulted in several immune-related significant GO terms and pathways unique to this group, such as “autophagosome”, “cytosolic DNA-sensing pathway” and “response to type I interferons”. Understanding how experimental functional feeds can impact the host response and the trajectory of co-infections will be an essential step in identifying efficacious intervention strategies that account for the complexities of disease in open cage culture.

## Introduction

Atlantic salmon (*Salmo salar*) is an economically important protein source with an estimated annual aquaculture production exceeding 2.24 million metric tonnes globally in 2016 (1). However, various ongoing diseases have strongly threatened the salmonid industry and resulted in significant economic losses. Among them, sea lice, which are ectoparasitic copepods, continue to cause notable damage to the salmonid farming industry around the world (2). Sea lice feed on the epidermis (mucus and skin) of the fish upon attachment and increase blood components in their diet as they smolt from molts from sessile stages to mobile pre-adults and adults (3). These latter stages cause damage to the host resulting in decreased growth and/or secondary infection. During the host-lice interaction, it has been observed that sea lice resistance is associated with acute host inflammation, and secretory products of lice exert immune-modulatory effects on the fish host (4).

Infectious salmon anemia virus (ISAv) is a World Organization for Animal Health (OIE)–listed orthomyxovirus that can cause as high as 90% mortality in infected Atlantic salmon (5). The virus typically spreads horizontally through the exposure of naïve fish to infectious material through the water column, contaminated equipment, or coprophagy. The infection’s primary target tissues are kidney, liver, and spleen (6). In the net-pen culture of Atlantic salmon, identification of ISAv positive fish often occurs in fish co-infected with sea lice (7), and lice infection has been found to down-regulate inflammatory signals and cell-mediated immune responses (4, 5, 8). This type of interaction of one pathogen (e.g., sea lice) with the host immune system, can alter the pathogenesis and progression of another pathogen (e.g., ISAv) under the co-infection scenario (9). Moreover, it has been reported that the efficacy of vaccines to the bacterial pathogen *Piscirickettsia salmonis* can also be largely reduced during co-infection with lice (*Caligus rogercresseyi*) (10). Thus, it is expected that co-infections could modulate the host response biochemically and transcriptionally, leading to dramatically different clinical outcomes compared to single infections.

Traditional intervention with parasiticides has led to the increased chemical drug resistance in sea lice (11, 12). As an alternative, functional feeds have been considered as an effective application to improve the population’s general health status and reduce the risk of disease by modulating the host immune system and its response to sea lice. Immunostimulants [often pathogen-associated molecular patterns (PAMPs) that initiate innate immune responses] are substances that activate the animal’s immune system for the prevention of diseases and improvement of the body’s natural resistance to various viral and bacterial infections. Sutherland et al. (13) found that a functional feed containing certain levels of peptidoglycan and nucleotide formulations successfully reduced the total *Lepeophtheirus salmonis* burden by 50% relative to fish fed a control diet. Functional feeds are already being used frequently in Atlantic salmon aquaculture and found to promote Atlantic salmon’s growth, improve their immune system, and induce physiological benefits beyond traditional feeds (14). Additionally, modulation of the fatty acid composition can also affect the host immune system. For example, vegetable oils contain a limited level of omega-3 long-chain polyunsaturated fatty acid (ω3-LC-PUFAs) such as eicosapentaenoic acid (EPA) and docosahexaenoic acid (DHA). Simultaneously, they have comparatively high levels of omega-6 fatty acid (ω6 FAs), which can drive pro-inflammatory responses and exert a negative impact on the fish (15). Therefore, we aimed to investigate the impact of ω3 and ω6 FA content in functional feed on skin tissue transcript expression in response to single infection and co-infection.

In the current study, whole transcriptome analysis was used to evaluate the impact of four experimental functional feeds (i.e., 0.3% EPA/DHA+high-ω6, 0.3% EPA/DHA+high-ω6+ immunostimulant (IS), 1.0% EPA/DHA+high-ω6, and 1.0% EPA/DHA+high-ω3) on uninfected Atlantic salmon (*S. salar*), during a single infection with sea lice (*L. salmonis*), and a co-infection with sea lice and ISAv. The study also aimed to identify and catalogue diet-specific molecular biomarkers and their respective immune response in three group treatments (i.e., uninfected, lice infection, and co-infection groups). Our study provides an improved understanding of the mechanisms and pathways underlying the host response during both lice infection and co-infection, that are important for identifying efficacious intervention strategies that account for the complexities of diseases in open cage culture.

## Materials and Methods

### Experimental Diets

All four experimental diets were formulated by Cargill Innovation Center (Dirdal, Norway) stemming from a standard salmon diet formulation. Experimental diets were as follows: Experimental diet 1 (0.3% FA): 0.3% EPA/DHA and high ω6; Experimental diet 2 (0.3% FA+IS): same as diet 1 with the addition of an immunostimulant; Experimental diet 3 (1% FAω6): 1% EPA/DHA and high ω6; Experimental diet 4 (1% FAω3): 1% EPA/DHA and high ω3. The details of the composition of four diets are provided in **Table 1**. Three of the diets were the same as used in Katan et al. (16) and the relationship was indicated in **Table 1**. The four diets were initially blinded to investigators until the completion of the trial.

**Table 1:** Experimental functional diet composition.

	0.3% FA(0.3% EPA+DHA High Ω6)^a^	0.3% FA+IS(0.3% EPA+DHA High Ω6 Immunostimulant)	1% FAω6(1% EPA+DHA High Ω6)^b^	1% FAω3(1% EPA+DHA High Ω3)^c^
Fish oil (% diet)	0.09	0.09	4.32	4.25
Soy oil (% diet)	12.50	12.50	10.10	–
Linseed oil (% diet)	–	–	–	6.45
Poultry fat (% diet)	2.41	2.41	0.58	4.30
Rapeseed oil (% diet)	–	–	–	–
Added oil (% diet)	15.00	15.00	15.00	15.00
EPA+DHA (% diet)	0.34	0.34	1.00	1.00
Saturated (% total FA)	17.0	17.0	17.0	17.0
18:2n-6 (% total FA)	48.1	48.1	38.1	12.3
18:3n-3 (% total FA)	7.2	7.2	5.9	24.6
n-6 (% total FA)	0.3% EPA+DHA	0.3% EPA+DHA	1% EPA+DHA	1% EPA+DHA
n-3 (% total FA)	High Ω6	High Ω6	High Ω6	High Ω3

^a^this diet was the same diet indicated as 0.3% FA↑ω6 in Katan et al, 2020. ^b^this diet was the same diet indicated as 1% FA↑ω6 in Katan et al, 2020. ^c^this diet was the same diet indicated as 1% FA↑ω3 in Katan et al, 2020.

### Fish Husbandry and Experiment Design

Atlantic salmon (*S. salar*), Saint John River strain smolts (weight (mean ± SD): 90 ± 15 g) were obtained from Cooke Aquaculture Inc. and transferred to the Aquatic Biological Containment Level II Facility at the Atlantic Veterinary College (Charlottetown, PE). Upon arrival, the fish were stocked into 170 L tanks (n=36) at the density of 40 fish per tank, supplied with fresh well water at 10.5 ± 1°C in a single-pass system with a 14 h: 10 h light-dark photoperiod. Fish were anesthetized in tricaine methanesulphonate (TMS (Syndel, Nanaimo, BC, Canada), 150 mg/L), individually weighed, and injected intraperitoneally (I.P.) with a passive integrated transponder (PIT) tag (AVID, CA, US) for identification purposes. Following acclimation for two weeks, the system was transferred to a partially-closed, recirculating aquaculture seawater system and the fish were transitioned to artificial seawater (SW; Instant Ocean^®^, Spectrum Brands Canada Inc, IL, USA) by increasing the salinity by 2-3 ‰ per day until a salinity of 33 ± 2 ‰ was achieved. Water quality was monitored daily until parameters (ammonia-nitrogen: 0.00-0.05mg/L, nitrite-nitrogen: 0.00-0.15 mg/L, nitrate-nitrogen: 0-60 mg/L, pH: 7.8-8.5) were within the ideal range. Thereafter, the water quality was monitored twice weekly. During the acclimation period and transition to SW, fish were fed daily at 1% body weight with EWOS Transfer (Surrey, BC, Canada). Following one week of acclimation to SW at 33 ± 2 ‰, four experimental diets were randomly assigned to 32 tanks (8 tanks per diet). Fish were continued to be fed at 1% body weight per day with the daily ration split between two feeding periods. Feed consumption was assessed using a feed scoring system (17), and individual tank effluent was flushed out of the tank just prior to and within one hour after each feeding.

### Single Infection (*L. salmonis*) and Co-Infection (*L. salmonis* and ISAv)

**Figure 1:**
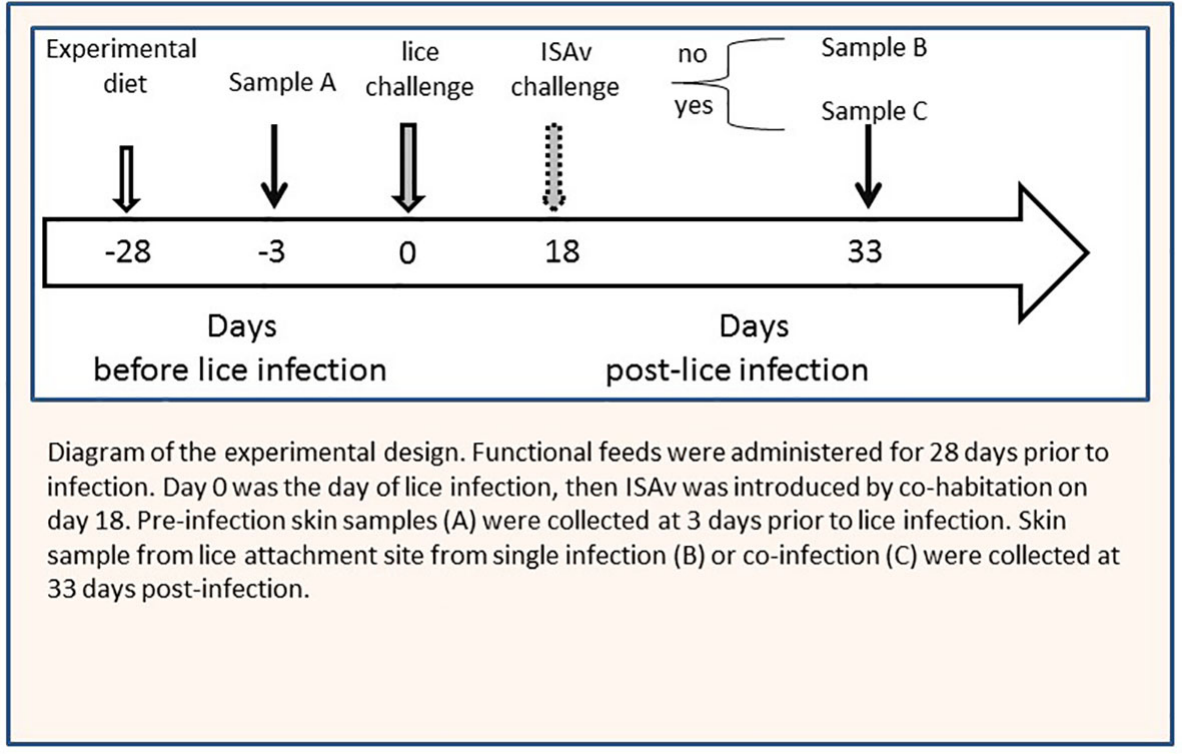
Diagram of the experimental design. Experimental diets were administered for 28 days prior to infection. Day 0 was the day of lice infection, then ISAv was introduced by co-habitation on day 18. Pre-infection skin samples sample A were collected at 3 days prior to lice infection. Skin sample from lice attachment site from single infection sample B or co-infection sample C were collected at 33 days post-infection.

After 28 days of the above feeding regime, all 16 tanks were infested with sea lice (*L. salmonis*) copepodids provided by the Huntsman Marine Science Centre (St. Andrews, NB, Canada). Prior to exposure, water flow was turned off to all tanks, and the water level was reduced below outflows. Fish were challenged at 50 copepodids/fish. Supplemental oxygen was added during the infection procedure to maintain 6.0-9.0 mg/L O_2_ for the 2-h exposure period. Lice-exposed fish exhibited behaviors associated with lice infections, including flashing, rubbing, and jumping throughout the exposure. After the 2-h infection, water flow was restored. The ISAv isolate (ISAV-HPR4 RPC/NB 04-085-1) used in the co-infection was provided by Fisheries and Oceans Canada, Moncton, NB. The prepared high-virulence ISAv isolate, harvested from Atlantic salmon head kidney tissue (18), was suspended in L-15 culture media. The ISAv isolate was grown in ASK cells (culture medium: L-15 media (Wisent Inc, Saint-Jean-Baptiste, QC, Canada) + 2% fetal bovine serum (FBS; Wisent Inc), penicillin/streptomycin, and fumagillin; Gibco, Grand Island, NY, USA) using the Spearman-Kärber method (19). Viral isolates were stored at -80°C in 0.5-mL aliquots until use. At 9 days post-lice infection, naïve donor fish (n=160; 40 fish/tank) were anesthetized (TMS: 150 mg/L) and I.P. injected (100 µl) with a 1 × 10^4^ TCID_50_ (Median Tissue Culture Infectious Dose) of the ISAv isolate. Donor fish were maintained for 7 days post-injection in a separate recirculation system to allow shedding of viral particles from these fish before adding to the tanks that were receiving the experimental diets (5 donor fish per tank) at 18 days post-infection (dpi) (20). Fish were monitored three times daily and moribund or dead fish were removed upon observation. Fish (2 fish/tank; n=64) from each experimental diet group and infection regimes (lice infection and co-infection) were sampled at 3 days prior to challenge with *L. salmonis* (i.e., pre-challenge control group). Ten fish/tank (40 fish/experimental diet) were opportunistically selected and euthanized by TMS overdose (250 mg/L) at 33 dpi when mortalities first appeared in cohabitants. Fish weight and full body-length were recorded. The sea lice load in each fish was quantified, and posterior kidney samples were collected to determine viral load. Skin samples were collected at 3 days prior to infection and 33 dpi (at louse attachment sites) from each fish (**Figure 1**). These samples were flash-frozen and stored at -80°C for RNA sequencing (RNA-seq) analysis. All procedures involving the handling and treatment of fish used were conducted in accordance with the UPEI Animal Care Committee (Protocol # 16-051).

### RNA Extraction, Library Construction and Sequencing

Total RNA was extracted from skin samples with a bead-based system from 6 representative fish from each feed group within each infection regimen using Trizol reagent (Ambion, CA, USA), according to the manufacturer’s instructions. RNA concentration and purity were estimated using the NanoDrop 2000 spectrophotometer (Thermo Scientific). RNA samples with an A260/A280 from 1.8 to 2.0 and an A260/A230 from 2.0 to 2.3 were used for the subsequent analyses. Total RNA was column-cleaned (Qiagen) with DNase treatment and sent to the Center for Aquaculture Technologies (PEI, Canada) for RNA-seq library preparation and sequencing. RNA integrity was further examined using the Bioanalyzer (BioRad) and only samples with RIN >7.5 were included for further analysis. cDNA libraries were prepared using the TruSeq RNA Sample Preparation Kit (Illumina) following the manufacturer’s protocol. Sequencing was conducted using the Hiseq 2000 (Illumina) platform with 200 bp paired-end reads. The raw reads were deposited in the NCBI’s Sequence Read Archive (SRA) under Accession No. PRJNA705415.

### RNA-Seq Data Analysis

The raw reads were trimmed of adaptor sequences and low quality reads were filtered out using Trimmomatic v0.32 (21). Illumina-specific adaptors were clipped from reads and the reads with an average Phred score less than 20 were trimmed. The processed reads were mapped to the Atlantic salmon reference genome release 100 (GenBank Accession No. GCF_000233375.1), and we further mapped processed reads and identified splice junctions using TopHat package v2.1.1 (22). The mapped reads resulting from TopHat were subjected to Cufflinks program v2.2.1 for transcript assembly and expression quantification. The output files were analyzed by Cuffdiff program v2.2.1 to identify the differentially expressed genes (DEGs) (23). The visualization and downstream analysis were conducted using R packages CummeRbund v2.34.0 (24) and clusterProfiler v3.18 (25). Genes with an adjusted *p*-value < 0.05 and log_2_ |fold-change| >1 were defined as significantly differentially expressed in this study. Genes with multiple gene annotations in Cuffdiff result column ‘gene’ and rows with fold-change values marked as ‘-inf’ or ‘-nan’ were excluded from the downstream analysis. Furthermore, genes with unknown annotation against the reference annotation file were filtered away for gene ontology analysis.

### Gene Ontology and Pathway Analysis

Gene ontology (GO) term enrichment analysis of DEGs was performed using R package clusterProfiler v3.18 (25). The annotation required for clusterProfiler analysis was prepared using R-based AnnotationHub for *S. salar* database (database number: AH207) (26). The DEGs were also analyzed by ClueGO v2.5.7 (27) available from Cytoscape v3.8.2 (28) plug-in tools to decipher functionally grouped significant gene ontology and KEGG (Kyoto Encyclopedia of Genes and Genomes) pathway annotation network from *Salmo salar* organism. ClueGO analysis was performed using default values, i.e. enrichment/depletion (two-side hypergeometric) test for terms/pathways with kappa score 0.4. *P*-values were corrected for multiple testing using Bonferroni step down method. To obtain unique gene lists for GO analysis in the diet-responsive study of pre-infected samples, the mean expression of each significant gene was obtained, and the genes were categorized into each diet according to the ascription of their highest expression level. The resulting GO terms and pathways with adjusted *p*-value only (False discovery rate (FDR) > 0.05) are presented and discussed in the current study.

### Quantitative Real-Time PCR (qPCR) Validation

A total of 11 genes (i.e., 9 genes of interest and 2 reference genes) were selected for qPCR analyses with gene-specific primers designed using Primer 3-based online design tool Primer-BLAST on NCBI (29, 30). The selected genes and corresponding primers used for validation are presented in **Supplementary Table 1**. Total RNA was extracted using Trizol reagent and cleaned with RNeasy Plus Universal Mini kit including the DNase treatment step (Qiagen). The cDNA templates for qPCR were synthesized in a 30-μL reaction using 1.5 μg of extracted total RNA using ThermoFisher’s High Capacity cDNA kit as recommended by the manufacturer’s instructions. Primer efficiencies were evaluated by generating transcript-specific standard curves (5-point, 3-5 fold serial dilution) using a pooled template prepared by combining 5 μL cDNA aliquot of each study sample (31) and found to be between 86 - 106%. Melt curves showed single product formation and absence of primer dimers for all transcripts tested. The qPCR protocol was as described previously (32). Specifically, the qPCR was conducted on CFX96 Touch Real-Time PCR System (Bio-Rad), using the following thermal program: initial activation of 95°C for 30 s, 95°C for 15 s then 60°C for 30 s (40 cycles), followed by a melt curve analysis from 65 to 95°C with fluorescence being read every 0.5 s with a ramp rate of 0.5°C. Each reaction (10 μL) consisted of 5 μL of Sso Advanced™ Universal SYBR Green Supermix (Bio-Rad), 2 μL of cDNA template, 2 μL of nuclease-free water (Bio-Rad), and 0.5 μL of both forward and reverse primers (10 μM as working concentration). Each sample was run in triplicate, and no-RT (no reverse transcriptase) controls and no-template control (NTC) were also included for each assay.

The qPCR data were analyzed using Maestro (BioRad), and calibrated normalized relative quantities (CNRQ) were calculated. Two endogenous reference genes, *eif* and *rps20*, were used and they were normalized with an M-value of 0.26 (33). The triplicate deviation maximum allowed for inclusion in the analysis was set at 0.50 Ct. One-way analysis of variance (ANOVA) was performed with R base package to determine the significance (*p* < 0.05). Tukey’s HSD test was used to determine the groups with significantly different expression profiles if significant results were detected.

## Results

### Feed Trial and Challenge

Atlantic salmon smolts were fed at 1% body weight per day for the duration of the study (**Figure 1**). There was no difference in feed consumption between the experimental diet groups, but feed consumption was significantly lower in the co-infection compared to the lice infection alone in all diets between 15 and 34 dpi (Results described in the companion paper (34, submitted). No significant differences were observed in body weight or length among the different diet treatments before the sea lice challenge (data not shown). Lice counts in the 1% FAω3 diets were significantly lower than those in the 0.3% FA diets by the end of the study (47 dpi) but only in the single infection group. And viral load was highest (lowest average Ct) in the 1% FAω3 diet, whereas the viral load was lowest (highest average Ct) in the 0.3% FA+IS diet (**Table 2**). Viral load, however increased in all groups by 47 dpi, with the highest load being present in the 0.3% FA diets (34, submitted). There were < 5% mortalities in fish infested with the single *L*. *salmonis* infection, compared to > 35% in all co-infected groups. The 0.3% FA diet yielded a better cumulative survival rate (47.8%) compared to the 1% FAω3 diet (37.3%) during the co-infection, and the addition of immunostimulant to the 0.3% FA diet i.e., 0.3% FA+IS diet further improved the survival rate (50.0%) in Atlantic salmon (**Table 2**). However, despite these improvements, diet did not significantly impact the mortality rate of co-infected fish. Both the diets containing 1% EPA/DHA (1% FAω6 and 1% FAω3) had lower survival rates compared to the 0.3% EPA/DHA diets (only the 1% FAω3 had a significantly lower survival) (34, submitted).

**Table 2:** Average lice counts and viral load (mean ± SD) of fish exposed to a single (lice) and co-infection (lice-then-ISAv) and cumulative survival rate for co-infection.

Name	Diet	Average lice counts			Virus load(Ct)	Cumulative survival rate (%)
		Pre-Infection	Single Infection(33dpi)	Co-Infection(33 dpi)	Co-Infection(33 dpi)	
0.3% FA	0.3% EPA/DHA+high-ω6	0 ± 0	12.9 ± 0.8	11.7 ± 0.9	32.9 ± 1.5	47.8
0.3% FA+IS	0.3% EPA/DHA+high-ω6 + immunostimulant	0 ± 0	11.2 ± 0.7	14.1 ± 1.0	33.3 ± 0.9	50.0
1% FAω6	1% EPA/DHA+high-ω6	0 ± 0	12.0 ± 1.0	13.6 ± 1.1	32.2 ± 0.2	46.7
1% FAω3	1% EPA/DHA+high-ω3	0 ± 0	10.4 ± 0.8	16.1 ± 0.9	31.8 ± 0.7	37.3

### Skin Transcriptomic Response by Single Infection and Co-Infection

**Figure 2:**
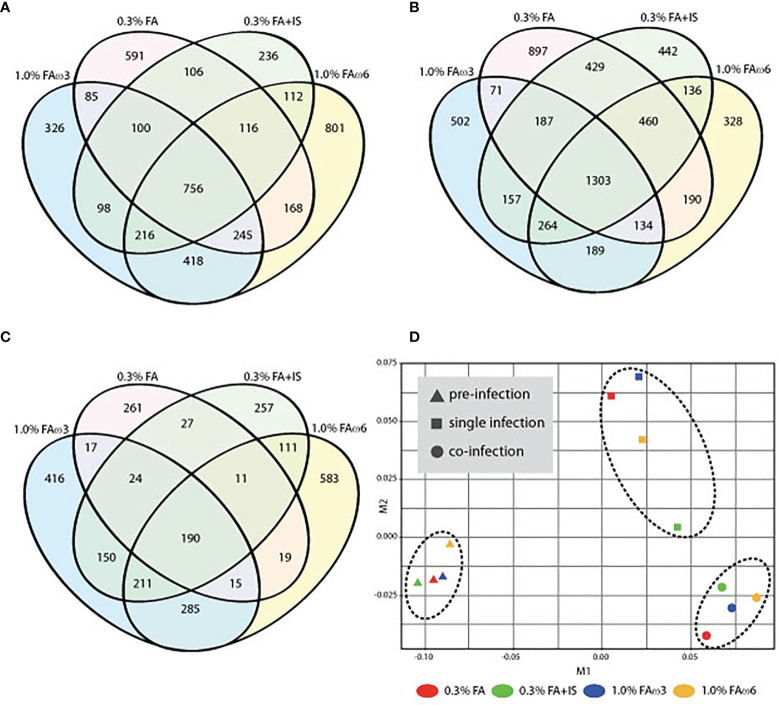
Overview of the global transcript expression profiles. **(A)** Venn diagram showing the distribution of DEGs identified between single infection and pre-infection among the four different diets. **(B)** Venn diagram showing the distribution of DEGs identified between co-infection and pre-infection among the four different diets. **(C)** Venn diagram showing the distribution of DEGs identified between co-infection and single among the four different diets. **(D)** Multidimensional scaling (MDS) analysis of the expression data similarity among the 12 groups.

To explore the transcriptional response in the skin (at sea lice attachment site) by lice infection alone and co-infection (at 33 dpi), the DEGs of the group of interest were identified using Cuffdiff, and the results are presented in **Tables 3**, **4**. In total, there were 756 (280 up-regulated and 476 down-regulated) and 1303 DEGs (649 up-regulated and 654 down-regulated) shared among the four experimental diets when comparing single infection groups and co-infection groups to control (pre-infected) groups, respectively (**Figures 2A, B**). We also identified 190 DEGs (186 up-regulated and 4 down-regulated) when comparing single and co-infection groups (**Figure 2C**). Multidimensional scaling (MDS) analysis segregated the samples based on infection challenge (i.e., pre-infection, single infection, and co-infection) and not from the dietary regimen, indicating that the infection challenge had a greater impact on the grouping than the diet (**Figure 2D**).

**Table 3:** Comparison of differentially expressed genes (DEGs) within each diet group.

Diet	Treatment Comparison	Number of DEGs		
		Up-Regulation	Down-Regulation	Total
0.3% FA	Single infection vs Control	955	1289	2244
	Co-infection vs Control	1638	1169	2807
	Co-infection vs Single infection	1016	292	1308
0.3% FA+IS	Single infection vs Control	976	1191	2167
	Co-infection vs Control	1482	2189	3671
	Co-infection vs Single infection	276	288	564
1.0% FAω6	Single infection vs Control	589	1151	1740
	Co-infection vs Control	1662	1716	3378
	Co-infection vs Single infection	865	116	981
1.0% FAω3	Single infection vs Control	1221	1611	2832
	Co-infection vs Control	1567	1437	3004
	Co-infection vs Single infection	1042	383	1425

False discovery rate (FDR)<5%.

### DEGs Under Different Experimental Diets Before Challenge

**Figure 3:**
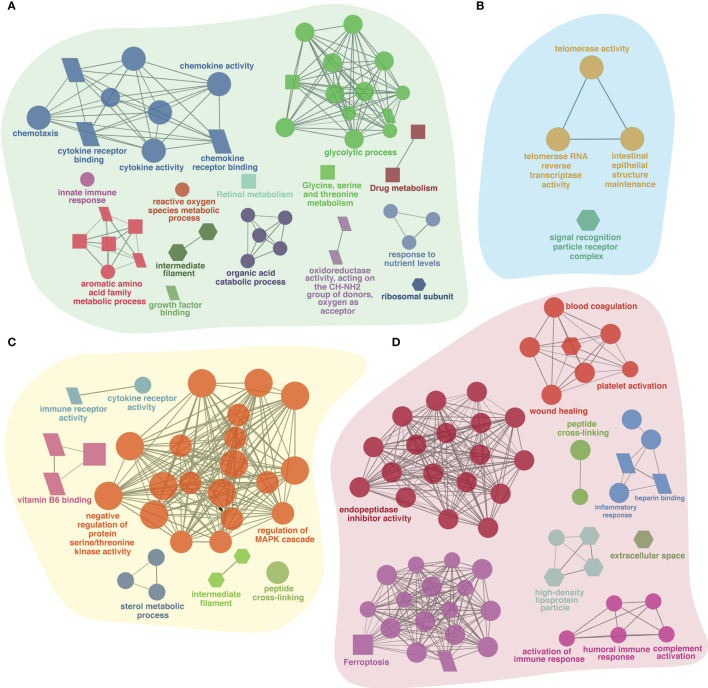
The ClueGO based enriched gene ontology (GO) terms and pathway identified from genes differently experessed in four experimental diets **(A)** 0.3% FA+IS, **(B)** 1% FAω3, **(C)** 1% FAω6, and **(D)** 0.3% FA of pre-infected group of samples. The shape size shows the GO terms and pathway significance (larger shape (e.g. circle, square) = higher significance). The shape depicts database source i.e., GO biological process (circle), GO cellular component (hexagon), GO molecular function (parallelogram), and KEGG pathways (square). The statistics of representative GO terms or pathways are tabulated in **Supplementary File 2**.

Prior to infection, fish receiving different experimental diets for 28 days exhibited varying global transcriptomic profiles in the dorsal skin tissue. The PCA analysis highlighted the greatest observed difference for the diet containing immunostimulant (i.e., 0.3% FA+IS) compared with all the other diets, and the transcriptomic profiles of fish administered with the two fatty acid-enriched diets (1% FAω3 and 1% FAω6) were characterized closer (**Supplementary Figure S1**). The numbers of significant DEGs identified among the four diets between pre-challenge samples are shown in **Table 4** (see **Supplementary File 1** for DEGs details and statistics). The heatmap of the DEGs with the four diets prior to infection did not show a well-grouped cluster, indicating a moderate effect of the diet manipulation at the skin transcriptome (**Supplementary Figure S2**). The 0.3% FA+IS diet significantly modulated the “chemotaxis”, and “cytokine/chemokine receptor binding” in the innate immune response (**Figure 3A**). The significantly upregulated genes of pro-inflammation in fish fed with the immunostimulant diet (0.3% FA+IS) compared to the control diet include C-C motif chemokine 19-like (*ccl19*), C-C motif chemokine 20-like (*ccl20*), C-C motif chemokine 4-like (*ccl4*), interferon-induced protein with tetratricopeptide repeats 5-like (*ifit5*), and interferon-stimulated gene 15 (*isg15*; **Supplementary File 1**, sub-table T1). On the other hand, the increased FA promoted the sterol metabolism, vitamin uptake and signal transduction in the immune response (**Figures 3B, C**). Surprisingly, the 0.3% FA diet, which was considered as control diet for this study, promoted the innate immune responses by elevated pathways in “ferroptosis”, “complement activation”, and “inflammatory response” (**Figure 3D** and **Supplementary File 2**).

**Table 4:** Comparison of differentially expressed genes (DEGs) among the diet groups.

Treatment	Diet Comparison	Number of DEGs		
		Up-Regulation	Down-Regulation	Total
pre-infection	0.3% FA+IS vs. 0.3% FA	217	158	375
	1% FAω6 vs. 0.3% FA	79	68	147
	1% FAω3 vs. 0.3% FA	27	69	96
	1% FAω6 vs. 1% FAω3	77	28	105
	0.3% FA+IS vs. 1% FAω3	110	68	178
	1% FAω6 vs.0.3% FA+IS	146	157	303
single infection	0.3% FA+IS vs. 0.3% FA	445	37	482
	1% FAω6 vs. 0.3% FA	70	40	110
	1% FAω3 vs. 0.3% FA	52	71	123
	1% FAω6 vs. 1% FAω3	103	48	151
	0.3% FA+IS vs. 1% FAω3	725	233	958
	0.3% FA+IS vs.1% FAω6	537	27	564
co-infection	0.3% FA+IS vs. 0.3% FA	63	244	307
	1% FAω6 vs. 0.3% FA	220	351	571
	1% FAω3 vs. 0.3% FA	139	238	377
	1% FAω6 vs. 1% FAω3	204	113	317
	0.3% FA+IS vs. 1% FAω3	104	149	253
	1% FAω6 vs. 0.3% FA+IS	116	55	171

False discovery rate (FDR)<5%.

### DEGs Under Different Experimental Diets Under Lice Alone Infection

In the single lice infection, the majority of DEGs were up-regulated in the 0.3% FA+IS diet compared to the other three diets (445-725 up-regulated genes; **Table 4**; **Supplementary File 3**, sub-Table T1, T5, and T6). Compared to the 0.3% FA diet, the IS additive diet stimulated pathways involved in heat shock proteins, glycolysis, mucus production, and skeletal muscle development, while it suppressed genes of innate immunity such as interferon-induced protein 44-like (*ifi44*), *isg15*, *ccl4* and *fth* in the single infection (**Supplementary File 3**, sub-table T1).

### Shared Differentially Expressed Genes Across Diets During Lice Infection Alone (Single Infection vs. Pre-Infection)

In general, there were altogether 756 shared DEGs (280 up-regulated and 476 down-regulated) among diets in the lice infection alone at 33 dpi compared to uninfected fish (3 days prior to infection) (**Figure 2A** and **Supplementary File 5**, sub-table T1). Examples of differentially regulated immune genes after lice alone infection included interleukins (*il17d*, *il7r*, *il2rb*, *il12b*, and *il6st*), chemokines (*ackr3*, *ackr4*, *ccr2*, *ccr6*, *ccr7*, *ccr9*, *cxcl12*, *ccl13*, *ccl17*, *ccl20*), metallopeptidases (*adam9*, *adam17*, *adamts8*, *adamts12*, *adamts17*, *adamts18*, *adamts20*, *mmp11*, and *mmp15*), transcription factors (*gata3, stat1*), and apoptosis (*rnf213, map3k11*, *litaf*, *scarb2*, *tagap, lgals4*, and *bok*) (**Supplementary File 5**, sub-table T1).

**Figure 4:**
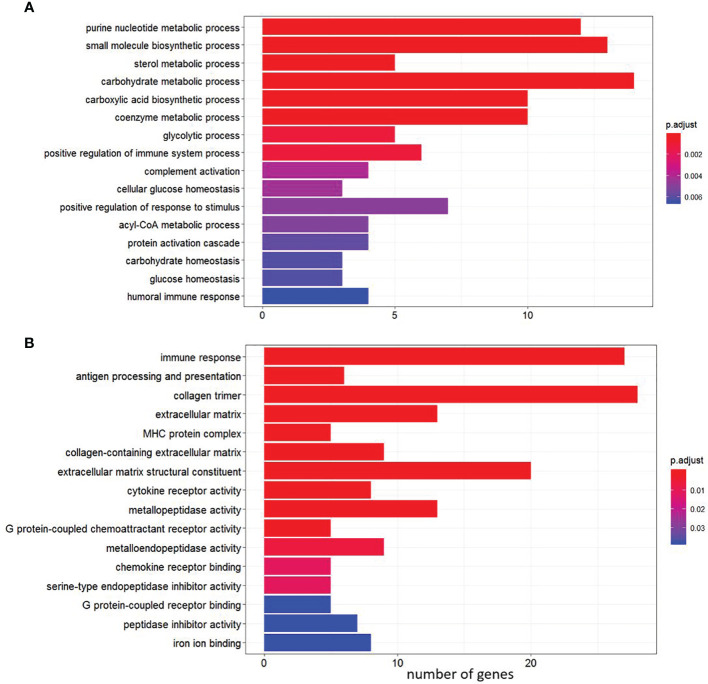
Gene Ontology (GO) pathway enrichment analysis of the shared genes among the four diets between single infection and control (pre-infection). **(A)** Enriched pathways among up-regulated genes. **(B)** Enriched pathways among down-regulated genes.

GO over-representation analysis of the up-regulated genes revealed enrichment of biological processes involved in a number of physiological functions including “glycolysis process”, “complement activation” and “sterol metabolic processes” (**Figure 4A**). In contrast, enrichment of GO terms represented by down-regulated DEGs included processes such as “antigen processing and presentation”, “collagen trimer”, “MHC protein complex”, “chemokine receptor activity” and “metallopeptidase activity” (**Figure 4B** and **Supplementary File 5**, sub-table T2).

### DEGs Under Different Experimental Diets Under Lice and ISAv Co-Infection

Under the co-infection, only 20.8% of the DEGs were up-regulated in the immunostimulant diet compared to 0.3% FA diet (**Supplementary File 4**, sub-table T1). The up-regulated genes were involved in skeletal muscle development (e.g., *myh2*, and *tnni2*), while the down-regulated genes were involved in multiple pathways, such as interferon activation, pro-inflammatory cytokine response, complement activation, and antigen-presentation (**Supplementary File 4**, sub-table T1). Compared to the 1% FAω3 diet, fish receiving 1% FAω6 diet and challenged with co-infection indicated significantly down-regulated gene expression in apoptosis and innate immune markers (e.g., *c3*, *c7* and *clec4e*), and up-regulated gene expression for heat shock proteins (e.g., *hspb1*, *hspb7*, and *hspb30*), and striated muscle development (e.g., *tnni2*, *myh2*; **Supplementary File 4**, sub-table T4).

### Shared Differentially Expressed Genes Across Diets During Co-Infection (Co-Infection vs. Pre-Infection)

In each diet, there were more DEGs in the co-infection than the sea lice alone infection (**Table 3**). A total of 1303 DEGs (649 up-regulated and 654 down-regulated) were identified as being shared across four diets in the co-infection (**Figure 2B**). The result of all the DEGs identified and reported in this section are listed in **Supplementary File 5**, sub-table T3. Selected DEGs with important biological roles are shown under different functional categories in **Table 5**. Substantial up-regulation in innate immunity was observed, which included genes in the interferon pathway (e.g., *ifi44, ifit5*, and *rsad2*) and complement system (e.g., *c4, c6* and *cd55*). Transcription of a large number of heat shock protein family members (e.g., *hspb7, hsp70-3, hspb8*) and apoptosis (e.g., *bag3* and *bcl2l13*) were also up-regulated, which indicated a stress response during the co-infection compared to the pre-infection. On the other hand, the transcription of genes involved in collagens (e.g*., col10a1, col11a1, col12a1*, and *col24a1*), antigen processing and presentation (e.g., *mr1, b2m, h2-aa, h2-eb1*, and *rt1-b*), T-cell development (e.g., *cd28, cd5, tagap*, and *cd96*), and chemokine signaling (e.g., *ccl4, ccl20, ccr6*, and *cccr9*) were significantly down-regulated in the co-infection compared to pre-infection. This list also includes DExD-Box helicase genes (*ddx6* and *ddx21*) that play important roles in general transcription, RNA editing, RNA transport and RNA biogenesis. Moreover, dual specificity phosphatases (such *dusp1*, *dusp4*, *dusp5*, and *dusp7*) were found up-regulated, which facilitates dephosphorylating MAP kinases that were involved in wide variety of cellular processes such proliferation, differentiation, transcription regulation. Furthermore, lipopolysaccharide-responsive genes such as *litaf*, *lrba*, and *lrbb* were also up-regulated in the co-infection. The *litaf* gene causes secretion of tumor necrosis factor alpha (TNF-alpha) and associated inflammatory mediators (adaptor proteins such as *lrba* and *lrbb*) that regulates the expression of various cytokines, endosomal protein trafficking, targeting proteins for lysosomal degradation and apoptosis. For pathway analysis, the down-regulated DEGs were enriched in pathways including “immune response”, “antigen processing and presentation”, “extracellular matrix”, “MHC protein complex”, “metallopeptidase activity”, and “iron ion transport” (**Supplementary File 5**, sub-table T4), while the up-regulated genes were enriched in pathways, such as “coenzyme biosynthetic process”, “sterol metabolic process” and “glycolytic process” (**Supplementary File 5**, sub-table T4).

**Table 5:** Selected list of differentially expressed genes (DEGs) during *L. salmonis* and ISAv co-infection in the skin of Atlantic salmon, when compared with the pre-infected control.

Category	Gene ID	Gene symbol	Gene description	Fold-changes (log_2_FC)			
				0.3% FA	0.3% FA+ IS	1% FAω6	1% FAω3
Muscle structure development	*fgf12*	*fgf12*	Fibroblast growth factor 12	3.31	3.54	4.02	3.70
	LOC106589658	*acta2*	Actin, alpha skeletal muscle 2	6.01	6.00	7.34	9.33
	LOC106575818	*myl3*	Myosin light chain 3	6.91	6.88	8.23	8.66
	LOC106609638	*tnnc1*	Troponin I	9.26	7.25	9.06	7.09
Interferon pathway	LOC106583433	*ifi44*	Interferon-induced protein 44	5.62	4.14	3.69	3.74
	LOC106608578	*ifit5*	Interferon-induced protein with tetratricopeptide repeat 5	5.80	3.15	5.80	5.57
	LOC106578964	*ifit5*	Interferon-induced protein with tetratricopeptide repeat 5	2.96	2.03	2.93	3.10
	LOC106566099	*rsad2*	Radical S-adenosyl methionine domain-containing protein 2	5.81	4.30	6.03	6.26
Complement system	*c6*	*c6*	Complement C6	4.52	4.81	3.28	4.36
	LOC106612870	*c4*	Complement C4	4.04	4.63	6.08	5.53
	LOC106572353	*cfh*	Complement factor H	2.12	2.29	2.41	2.04
	LOC106565874	*cd55*	Complement decay-accelerating factor	1.94	3.43	1.97	1.95
	*mbl2*	*mbl2*	Mannose-binding protein C	3.60	4.54	3.84	4.35
Heat shock proteins	*hspb7*	*hspb7*	Heat shock protein beta-7	7.27	8.27	5.07	6.61
	*hsp70*-3	*hsp70*	Heat shock protein 70	5.01	4.34	5.37	6.51
	LOC106603948	*hspb1*	Heat shock protein beta-1	4.64	3.30	5.69	3.18
	LOC106579825	*hspb8*	Heat shock protein beta-8	3.58	4.33	4.96	3.71
	*hspb8*	*hspb8*	Heat shock protein beta-8	1.90	2.13	2.31	1.73
Iron homeostatis	LOC106599278	*fth1*	Ferritin, middle subunit	-3.07	-1.94	-2.20	-1.84
	LOC106600764	*fth1*	Ferritin, middle subunit	-3.64	-1.59	-2.29	-2.39
Chemokine signaling	LOC106600142	*ccl20*	C-C motif chemokine 20	-2.89	-4.23	-2.47	-3.09
	*ccr6*	*ccr6*	C-C chemokine receptor type 6	-2.17	-2.93	-2.49	-2.16
	LOC106600446	*ccl4*	C-C motif chemokine 4	-2.14	-2.87	-2.19	-1.95
	LOC106590189	*ccr9*	C-C chemokine receptor type 9	-1.66	-2.92	-2.60	-3.10
Antigen presentation	LOC106564360	*h2-aa*	H-2 class II histocompatibility antigen, A-Q alpha	-2.17	-2.28	-2.67	-1.92
	LOC106600246	*h2-eb1*	H-2 class II histocompatibility antigen, I-E beta	-1.76	-3.26	-2.42	-2.04
	b2m	*b2m*	beta-2-microglobulin	-1.14	-1.82	-1.02	NS
	LOC106562659	*mr1*	Major histocompatibility complex class I-related gene protein	-1.69	-1.51	-1.78	-1.50
	LOC106564356	*rt1-b*	rano class II histocompatibility antigen, A beta chain	-1.78	-1.66	-2.03	-1.91
	LOC106565699	*h2-aa*	H-2 class II histocompatibility antigen, A-U alpha chain	-1.48	-2.95	-2.31	-1.64
T-cell development	LOC106586939	*cd28*	T-cell specific surface glycoprotein CD28	-2.14	-2.18	-1.83	-3.51
	LOC106602649	*cd5*	T-cell surface glycoprotein CD5	-1.73	-3.08	-2.27	-2.59
	*tagap*	*tagap*	T-cell activation Rho GTPase-activating protein	-1.52	-2.55	-2.10	-1.92
	LOC106563917	*cd96*	T-cell surface protein tactile	-1.42	-2.07	-2.12	-1.84
	LOC106611417	*trbc2*	T-cell receptor beta-2 chain C region	-1.36	-2.24	-1.85	-1.88
Collagen synthesis	*col11a1*	*col11a1*	Collagen alpha-1(XI) chain	-2.65	-4.68	-4.18	-4.20
	LOC106583145	*col12a1*	Collagen alpha-1(XII) chain	-2.15	-2.30	-2.26	-3.11
	LOC106593482	*col11a1*	Collagen alpha-1(XI) chain	-2.08	-2.96	-2.84	-3.07
	LOC106588396	*col11a2*	Collagen alpha-2(XI) chain	-1.72	-4.13	-2.89	-2.79
	LOC106607727	*col10a1*	Collagen alpha-1(X) chain	-1.66	-3.81	-3.44	-3.02
	LOC106584045	*col24a1*	Collagen alpha-1(XXIV) chain	-2.14	-3.59	-2.73	-3.18
Tissue repair	LOC100286414	*fmod*	Fibromodulin	-4.01	-5.28	-5.18	-4.44
	LOC106590496	*prg4*	Proteoglycan 4	-5.71	-5.90	-4.04	-5.12
	LOC101448046	*tmprss5*	Serine protease-like protein	2.40	NS	1.98	2.02
	LOC106562051	*mmp15*	Matrix metalloproteinase 15	-2.84	-2.88	-2.35	-3.13
	LOC106569443	*mmp14*	Matrix metalloproteinase 14	1.36	1.48	1.37	1.98
	fgfp12	*fgfp12*	Fibroblast growth factor 12	3.31	3.54	4.03	3.71
	fgfp1	*fgfp1*	Fibroblast growth factor-binding protein 1	2.55	3.89	3.71	3.36

NS, not significant; IS, indicates immunostimulant.

A comparison of the co-infection with the lice infection alone revealed most of the DEGs were up-regulated (186 up-regulated and 4 down-regulated) in the co-infection group (**Supplementary File 5**, sub-table 5). The enriched biological process pathways of the up-regulated genes in co-infection included “response to type I interferon”, “NAD biosynthetic process”, “innate immune response”, and “response to virus” (**Supplementary File 5**, sub-table T6).

### Hierarchical Clustering, GO Term and Pathway Enrichment of Shared Genes Identified in Pre-Infection, Lice Infection Alone, and Co-Infection Samples

**Figure 5:**
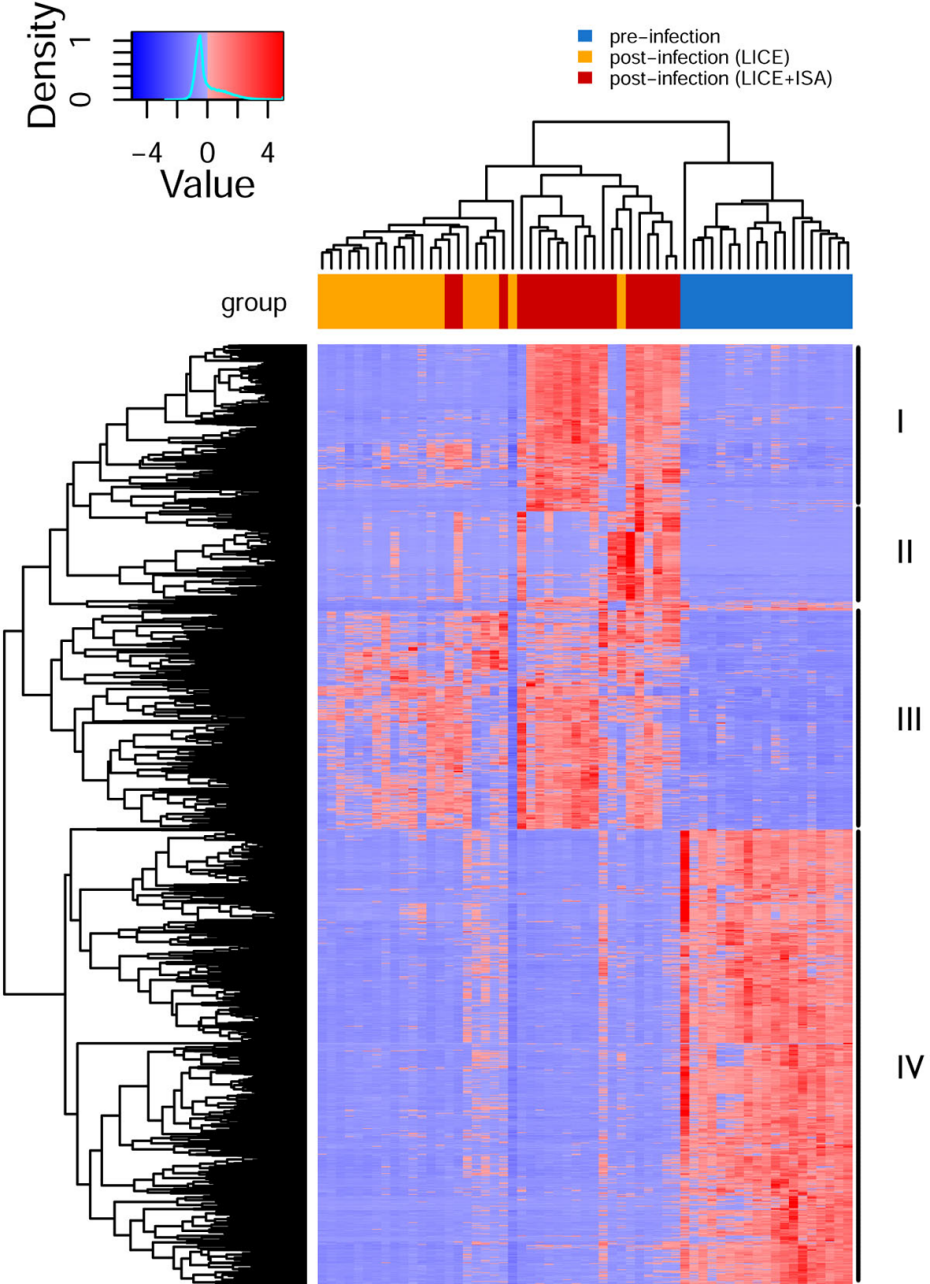
Hierarchical clustering of shared differentially expressed genes (DEGs) identified in three groups (single vs. pre-infection, co-infection vs. pre-infection, and co-infection vs. single infection). The normalized expression value (FPKM) for each samples (columns) and genes (rows) are illustrated in red (up-regulated) and blue (down-regulated) color in the heatmap.

In the current study, the shared genes identified in the three groups (i.e., pre-infection, lice infection alone, and co-infection with lice and ISAv; **Figure 2**) were combined and further subjected to agglomerative hierarchical clustering (complete-linkage clustering) using R package heatmap3 v1.1.9 in R version 4.0.2. The unsupervised clustering of the shared gene expression matrix resulted in three main clusters. The pre-infected samples grouped perfectly, while in the post-infected samples, clusters were represented by most samples from either lice infection or live+ISAv infection (**Figure 5**). This variation within the treatment groups was likely caused by the different viral load and infection stage in the co-infected samples during cohabitation. The clustering at gene level resulted in four major clusters. The cluster I, i.e., top 20% of heatmap consist of DEGs that were up-regulated in co-infection group compared with both other groups, e.g., *ifit5*, autophagy related protein 9A (*atg9a*), and *irf7b*. The smaller groups of genes in cluster II were mostly down-regulated in lice infection alone groups, pre-infection groups and half of co-infection group, e.g., *atp2a1*, DNA damage inducible transcript 4 like (*ddit4l)*, and bcl 2-like protein 13. The cluster III consists of approximately 370 up-regulated genes (**Supplementary Figure S3** for high resolution image) in lice alone and co-infection groups compared with pre-infection (**Figure 5**). The cluster IV represents the largest group of DEGs (about 780 genes) that were down-regulated in lice infection alone and co-infection groups compared with pre-infection (**Supplementary Figure S3**).

**Figure 6:**
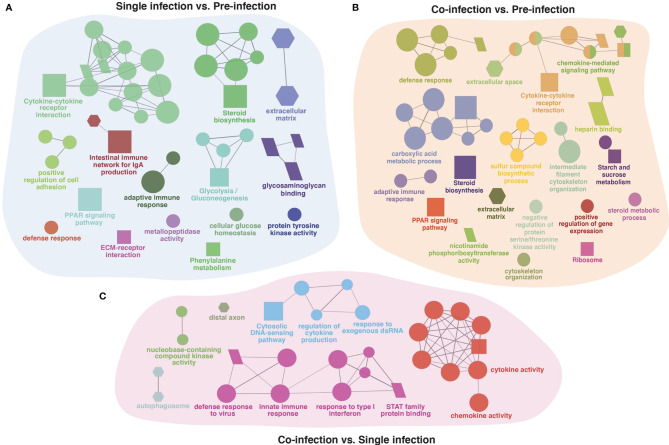
The ClueGO based enriched gene ontology (GO) terms and pathways identified from genes differently expressed in three group comparison. **(A)** single (lice) infection vs. pre-infection, **(B)** co-infection (lice+ISAv) vs. pre-infection, and **(C)** co-infection (lice+ISAv) vs. single infection. The shape size shows the GO terms and pathway significance (bigger he size higher the significance). The shape depicts batabase source i.e., GO biological process (ellipse), GO cellular component (hexagon), GO molecular function (parallelogram), and KEGG pathways (square). The statistics of representative GO terms or pathway are tabulated in **Supplementary File 7**.

The gene list obtained from shared DEGs among these three groups (pre-infection, lice infection, and lice+ISAv infection) was used for ClueGO analysis resulting in significantly enriched GO terms and pathways (adjusted p-value < 0.05; **Figure 6** and **Supplementary File 7**). The significant GO terms and enriched pathways identified in single infection vs. pre-infected samples consisted of various immune-relevant terms such as “defense response”, “steroid biosynthesis”, “PPAR signaling pathway”, “Cytokine-cytokine receptor interaction”, “extracellular matrix”, “metallopeptidase activity”, “Intestinal immune network of IgA production” and “adaptive immune response” (**Figure 6A**). Similar significant GO terms and pathways were enriched in co-infection vs. pre-infected samples in addition to “heparin-binding”, “negative regulation of protein serine/threonine kinase activity”, and “chemokine mediated signaling pathway” (**Figure 6B**). However, ClueGO analysis conducted between infected groups (single infection vs. co-infection) resulted in several immune-related significant GO terms and pathways unique to this group, such as “autophagosome”, “cytosolic DNA-sensing pathway”, “response to exogenous dsRNA”, “response to type 1 interferons” and “STAT family protein binding (**Figure 6C**).

### qPCR Validation

In order to validate gene expression values obtained by RNA-seq analysis, nine genes (4 up-regulated and 5 down-regulated genes) were selected for qPCR assay, including fibroblast growth factor-binding protein 1 (*fgfbp1*), *mbl2*, complement component C6 precursor (*c6*), *c4*, C-C chemokine receptor 6 (*ccr6*), *ccl4*, *ccr9*, T-cell surface glycoprotein CD5-like (*cd5*), and T-cell activation Rho GTPase-activating protein (*tagap*). Those genes were selected based on functional categories including would healing (i.e., *fgfbp1*), complement system (i.e., *c4*, *c6* and *mbl2*), B-cell differentiation (i.e., *ccr6* and *cd5*), T-cell regulation (i.e., *ccr9* and *tagap*) and inflammatory response (i.e., *ccl4*).

**Figure 7:**
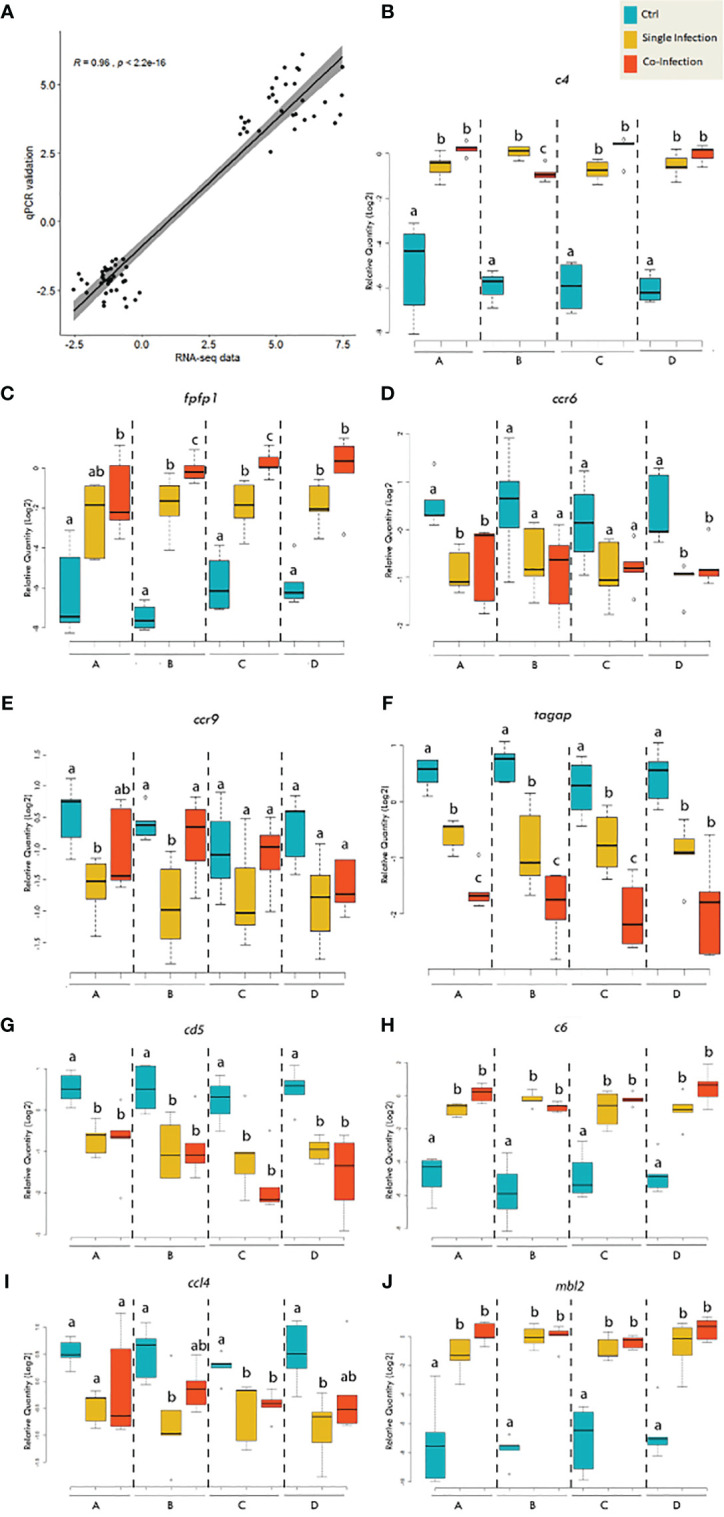
qPCR validation of the selected genes for RNA-seq data. **(A)** Scatterplot of log_2_-transformed gene expression fold-changes between treatment groups calculated from RNA-seq data and qPCR assay. **(B–J)** Boxplots of qPCR data for the selected genes of interest. Plots reveal median calibrated normalized relative quantities (CNRQs) values and interquartile ranges in log_2_ scale. On x-axis: letter A indicates the group receiving the 0.3% FA diet; letter B indicates the group receiving the 0.3% FA+IS diet; letter C indicates the group receiving 1% FAω6 diet; letter D indicates the group receiving the 1% FAω3 diet. Statistics was conducted within each diet. Different letters indicate significant difference (p<0.05).

Expression changes in these genes determined by qPCR were significantly correlated with those shown by RNA-seq (*R=0.96*; **Figure 7A**). In agreement with the RNA-seq result, qPCR results showed that both lice infection alone and co-infection significantly promoted the complement system indicated by transcript level of *c4, c6* and *mbl2*, although no significant differences were identified among dietary treatments (**Figures 7B, H, J**). Wound healing is a dynamic of extracellular matrix degradation and remodeling. Although pathway enrichment analysis from RNA-seq data indicated the suppressed expression of collagen synthesis, both qPCR and RNA-seq showed up-regulation of *fgfbp1* during the single and co-infection (**Figure 7C**). In addition, the qPCR analysis showed the chemokines *ccl4*, *ccr6*, *ccr9*, and *cd5* were significantly down-regulated during lice infection alone vs pre-infection (**Figures 7I, D, E, G**), and comparable with that of co-infection. The qPCR did not find significant changes in *tagap* transcription among dietary treatments, while the transcript level of *tagap* showed a substantial decrease from pre-infection (control), lice infection alone and co-infection in three diets (**Figure 7F**).

## Discussion

The mucosal barriers of skin in teleost fishes constitute the first line of defense against pathogen invasion. Here we investigated the effects of diet on host susceptibility to lice infection alone and lice-then-ISAv co-infection, and the host response at the louse attachment site on the skin using transcriptomic profiling. Result indicated that there was no statistically significant difference in mortality between the diets. However, the 0.3% FA diet yielded a better survival rate compared to the 1% FAω6 diet during the co-infection, and the addition of immunostimulant to the 0.3% FA diet (0.3% FA+IS) further increased the survival rate in Atlantic salmon. Transcriptomic analysis using RNA-seq in skin samples revealed that administration of diets containing immunostimulants for 28 days promoted a pro-inflammatory state prior to disease challenge. In addition, pre-exposure to sea lice in the co-infection may have compromised the host adaptive immune response through suppression of antigen processing and presentation, B and T-cell differentiation, and induction of a large cellular stress response. These factors may have contributed to ISAv susceptibility and mortality during the co-infection.

Fish mucosal secretions are known to contain a variety of antimicrobial peptides, complement proteins, proteases, and lysozyme (35). The mucosal secretions are an important strategy to protect against pathogen infections (36), and have been shown to be stressor-sensitive in teleost fish (37). It is well known that fasting causes the teleost host to be more susceptible to pathogen infection, and previous studies showed that this feed deprivation caused a rapid decrease in the density of epidermal mucous cells in Atlantic salmon (38) and catfish (39). After 28 days of feeding regime, we found the expression of *muc2* was significantly up-regulated in the 1% FAω3 diet compared to 0.3% FA diet. During lice infection alone, we also found that the fish fed with 0.3% FA+IS diet showed elevated expression of *muc2*, *muc5ac*, *muc*, and induced significant transcripts changes in iron homeostasis (e.g., ferritins) and pro-inflammatory immune response (e.g. chemokines *ccl4*, and *ccl20*) compare to 0.3% FA diet. In addition, fish fed with 1% FAω3 diet suppressed the expression of protein-glutamine gamma-glutamyl transferase (*tgm*; responsible for catalyzing the cross-linking proteins during formation of epidermis) indicating the impact of fatty acid-enriched diet on the maintenance of the dynamic of the skin structure. Taken together, our results showed that mucus properties appeared to shift in response to diets. This indicated that the changes in the mucus properties in the skin caused by nutritional stimulus could further affect the host-pathogen dynamics and their disease resistance in host fish.

Multiple functional feeds have been tested to prevent parasitic copepod infection in aquaculture. Infection with *L. salmonis* in Atlantic salmon was shown to be significantly reduced after 5 weeks of feeding glucosinolate-enriched diets, and this reduction was associated with up-regulation of host genes in skin tissue associated with type 1 pro-inflammatory factors, antimicrobial and acute-phase proteins, extracellular matrix remodeling proteases and iron homeostasis regulation (40). Sutherland et al. reported that *L. salmonis* infected Atlantic salmon fed an immunostimulatory diet containing a peptidoglycan and nucleotide formulation exhibited up-regulated expression of *il1b* in the skin and spleen (13). In addition, Covello et al. (41) demonstrated the effectiveness of incorporating CpG oligodeoxynucleotide or yeast extracts into post-smolt Atlantic salmon diets, resulting in a reduction in levels of *L. salmonis* by 40% in fish fed the immunostimulant compared to control with associated transient changes in inflammatory and extracellular matrix gene expression in the skin. The fish immune response to sea lice and other parasitic copepods has been reviewed by Fast (4) who reported a general association with early onset of skin inflammation at the attachment site, and implied that immunostimulatory feeds may protect the host against the types of immune-regulatory shifts that normally benefit the parasite. In our study, more than 93% of the DEGs were up-regulated in the fish fed with 0.3% FA+IS diet compared to 0.3%FA diet in the single lice infection. The immunostimulant diet increased the host’s basal energy metabolic rate, mucus production, and skeletal muscle structure development. However, in the case of co-infection, the majority (80%) of the DEGs were suppressed in the fish under 0.3% FA+IS regime, which included a broad array of immune-related pathways such as interferon activation, complement activation, pro-inflammation, and antigen presentation. It seems that the 0.3% FA+IS diet effectively boosted the mucosal immunity in the single lice infection, but this benefit was not maintained in the co-infection. Taken together, there appears to be a trade-off for better anti-parasitic responses in the co-infection scenario regarding to the diet selection.

The immunostimulant diets promoted a pro-inflammatory signature within the skin of Atlantic salmon and some of the up-regulated DEGs in the immunostimulant diet-fed group included *ccl19*, *ccl20* and *ccl4*. These chemokines not only promote leukocyte mobilization, but also regulate the immune response and differentiation of recruited cells. CCL19 in Ayu (*Plecoglossus altivelis*) was shown to promote a pro-inflammatory state, with a dramatically up-regulated M1-type monocytes/macrophages when challenged with *Vibrio anguillarum* (42). In mammals, CCL20 plays an important role in skin and mucosal surfaces under homeostatic and inflammatory conditions when it combines with c-c chemokine receptor CCR6, which activates a strong chemotactic response to attract dendritic cells (DC), effector/memory T-cells and B-cells at the site of infection (43). The functional role of *ccl4* has previously been examined in teleost and has been reported to be immediately up-regulated within 2 h of poly (I:C) or LPS stimulation. In addition, *ccl4* in orange-spotted grouper (*Epinephelus coioides*) was found to induce chemotactic activity in peripheral blood leukocytes, and up-regulate the gene expression of *tnf-α1, mx, ifn-γ*, indicating *ccl4* plays a role in promoting the inflammatory response and driving the lymphocyte differentiation towards the Th1 pathway (44). In our study, three copies of *ccl19* (paralogs) identified in the *S. salar* genome (*Salmo salar* Annotation Release 100), were all up-regulated after feeding with the immunostimulant diet, 0.3% FA+IS, compared with the 0.3% FA diet. There are a total of three paralogs of *ccl4* in the Atlantic salmon genome, where one of them (i.e., LOC106570886) was significantly up-regulated in the immunostimulant diet fed fish. The functional difference of these chemokine paralogues is unknown, however, this would suggest that some may have a redundant signaling function while, to a lesser extent, others may have a loss of function or a different function altogether (i.e. neofunctionalization).

The parasite initiates attachment to the host surface and causes wounds through mechanical and chemical actions. Cortisol is often produced and secreted systemically as a result of stress when parasites mature to the pre-adult life stage. Indeed, cortisol treatment has been shown to have a significantly greater impact on transcriptomic effects in Atlantic salmon than lice-induced changes. Cortisol alone stimulates the expression of genes involved in the metabolism of steroids and amino acids, and suppresses genes related to antigen presentation, B and T cell function, antiviral and wound healing responses (45). In our study, pathway enrichment analysis indicated that the glycolysis and sterol metabolic process were greatly promoted in the skin tissue of the fish at the site of attachment at 33 dpi. Interestingly, it is notable that, although the genes involved in fibroblast synthesis were significantly up-regulated, the genes involved in collagen synthesis were remarkably suppressed. The observed transcriptomic changes in our study, such as increased glycolysis, suppressed B and T cell differentiation, were largely in agreement with the outcome of cortisol upregulation. Although we did not measure the cortisol level in this study, it is well recognized that cortisol would be remarkably induced at the 33 dpi sample point, when lice have molted to pre-adult females and adult males (46). Cortisol has long been described as inhibiting collagen synthesis in mammals (47), and in our study the suppression of collagen synthesis coincided with the timing of an expected up-regulation of cortisol in the host. This would suggest that although *L. salmonis* immunomodulatory secretions are expected to have a localized impact on leucocyte chemotaxis, skin inflammation, and healing, the host response at the mobile louse attachment site appears to be heavily influenced by systemic stress and the downstream impacts of interrenal cortisol release.

The healing process in response to skin damage comprises a complex cascade of events including hemostasis, inflammation, cell proliferation, and tissue remodeling (45, 48). During the later stages of a healing response, wound contraction reduces the size of the tissue defect and subsequently decreases the amount of damaged tissue that needs to be repaired. During the wound healing process, fibroblasts synthesize the extracellular matrix and produce type I collagen (49) and differentiate into myofibroblasts which create the tensile force to pull the wound edges toward the wound center. Actin and myosin interact with the newly formed collagen fibers in the extracellular matrix, forming a web-like adhesive base for wound contraction, which results in gradual reduction of wound area (50). In our study, the tissue damage observed in the lice infection alone induced a proliferative response from the fibroblasts in the skin, as well as genes involved in skeletal muscle development and wound contraction, such as early growth response protein, fibroblast growth factor, fibroblast growth factor-binding protein, myosin, and actin filament. Myosin and troponins have previously been identified as responsive genes to sea lice attachment in salmon skin (51). Robledo et al. also observed that salmon susceptible to sea lice had a higher expression of genes in Atlantic salmon skin involved in muscle contraction, such as troponins and myosins, compared to salmon resistant to sea lice (52). The authors further proposed that the high lice burden in the susceptible fish provoked an increase in fish activity, which might be related to the up-regulation of muscle genes. Taken together, it appears likely that up-regulation of myofiber and muscle contractile proteins could be a result of a combination of wound contraction and physical stress response to lice burden.

Lice secretory products are known to cause profound changes in Atlantic salmon hosts at the site of attachment, including chemotaxis and signaling, antiviral response, redox homeostasis and major histocompatibility class I gene expression (8, 53). We found that while the innate immune system (e.g., complement system) was promoted in the lice infection, a large number of genes participating in antigen presentation and processing were significantly suppressed. Cellular immunity, activated by interferons (IFN) and other cytokines *via* antigen presentation through the MHC I pathway is critical in the host control of virus and parasitic infections. In addition, our study showed that transcript abundance of *cd4* (Th1 response), *cd209* (innate immune response), *ccr7* (Th2), and *il1b* (inflammation) were suppressed during the infection with sea lice alone. This indicates that lice suppress a variety of T cell regulation functions, including both Th1 and Th2 pathways. Similar to lice infection alone, genes involved in antigen processing and presentation were significantly down-regulated in the co-infection, while genes in complement activation were mostly significantly up-regulated. The overall immunosuppression, either caused directly by lice secretory/excretory products (8), or cortisol up-regulation from physiological stress caused by the lice, may make the fish host more susceptible to a secondary infection.

The mucosal immune system is the one of the largest components of the entire immune system (54). At onset of pathogen infection, innate immunity fulfills an important role in the body’s early defense against pathogen challenge, as well as initiates the acquired immune response. Type I interferons (IFN-α/β) protect other cells from further viral infection by binding to IFN-α/ß receptors, leading to induction of antiviral proteins such as Mx, ISG15 and protein kinase R (PKR) (55). During ISAv infection, the innate immune response included increased expression of Mx and ISG15 *via* an IFN-independent mechanism (56). Several studies describe TRIM proteins’ (e.g., *trim25* and *trim35*) antiviral functionality by enhancing IFN response against fish viruses (57–60). Various studies conducted in mammals and teleosts demonstrated TRIM proteins (*trim25* and *trim14*) are essential for RIG-I (*ddx58, dhx58, and cgas*) mediated antiviral activity (61–66). For the subsequent adaptive immune response, MHC class I and II molecules present antigenic peptides, to class I-restricted CD8+ T cells and class II-restricted CD4+ T cells, respectively. During ISAv infection, prior work has described up-regulated expression of MHC class-I, B2M, TRIM 25 and CCL19 (67, 68). Interestingly, transcript levels of genes related to MHC class II antigen presentation pathway and B lymphocyte responses have not been observed to change in studies of ISAv-infected fish. Barker et al. demonstrated that lice (*L. salmonis*) infected Atlantic salmon were more susceptible to ISAv, and exhibited reductions in MH class I and anti-viral genes (e.g., galectin 9, TRIM and ISGs, etc.) and similarly, Lhorente et al. reported that lice (*C. rogercresseyi*) reduced the resistance of Atlantic salmon to the bacterial pathogen *P. salmonis* (5, 69). In our study, transcriptomic comparison of the skin samples under co-infection vs pre-infection highlighted the up-regulated transcripts in innate immunity (e.g., IFN pathway, pro-inflammation, and complement system) and the stress response (e.g., heat shock proteins), and down-regulated transcripts in adaptive immunity and tissue repair. The transcripts of antigen presentation cells were significantly suppressed during the lice infection alone and subsequent co-infection. This interference of the antigen presentation and processing pathway by lice infection might be responsible for the host susceptibility to the secondary ISAv infection.

In summary, pre-exposure to *L. salmonis* increased the susceptibility of Atlantic salmon to the secondary infection of ISAv due to a compromised adaptive immune response, i.e. antigen presentation system and T cell differentiation. Our results provide baseline information to assist in deciphering the parasite-virus co-infection mechanism, and highlight the impact of dietary regime on modulating the mucosal immune events in teleost fish.

## Data Availability Statement

The datasets presented in this study can be found in online repositories. The names of the repository/repositories and accession number(s) can be found below: https://www.ncbi.nlm.nih.gov/, PRJNA705415.

## Ethics Statement

The animal study was reviewed and approved by UPEI Animal Care Committee (Protocol # 16-051).

## Author Contributions

Conceptualization, WC and MF. Data curation, WC and SK. Funding acquisition, MF, RT, and MR. Investigation, WC, SK, and MF. Methodology, MF, NG, TH, MA, UN, AC-S, and MR. Project administration, SW, SP, and LC. Validation, WC. Diet design, CP. Writing—original draft, WC. Writing—review and editing, SK, SW, UN, RB, MR, and MF. All authors have read and agreed to the published version of the manuscript.

## Funding

This work was part of the Integrated Pathogen Management of Co-infection in Atlantic Salmon (IPMC) project (Genomic Applications Partnership Program, GAPP #6607), funded by the Government of Canada through Genome Canada and Genome Atlantic. The project was also funded by Mitacs (through the Mitacs Accelerate program), and EWOS Innovation/Cargill Aqua Nutrition. The funder EWOS Innovation/Cargill Aqua Nutrition was not involved in the study design, collection, analysis, interpretation of data, the writing of this article or the decision to submit it for publication.

## Conflict of Interest

Authors RGT and RB were employed by the company Cargill Innovation.

The remaining authors declare that the research was conducted in the absence of any commercial or financial relationships that could be construed as a potential conflict of interest

## Publisher’s Note

All claims expressed in this article are solely those of the authors and do not necessarily represent those of their affiliated organizations, or those of the publisher, the editors and the reviewers. Any product that may be evaluated in this article, or claim that may be made by its manufacturer, is not guaranteed or endorsed by the publisher.

## References

[1] Fao. The State of World Fisheries and Aquaculture. Italy: FAO (2018).

[2] Costello MJ. The Global Economic Cost of Sea Lice to the Salmonid Farming Industry. J Fish Dis (2009) 32:115–8. doi: 10.1111/j.1365-2761.2008.01011.x 19245636

[3] Heggland EI Dondrup M Nilsen F Eichner C. Host Gill Attachment Causes Blood-Feeding by the Salmon Louse (Lepeophtheirus Salmonis) Chalimus Larvae and Alters Parasite Development and Transcriptome. Parasit Vectors (2020) 13:225. doi: 10.1186/s13071-020-04096-0 32375890 PMC7201535

[4] Fast MD. Fish Immune Responses to Parasitic Copepod (Namely Sea Lice) Infection. Dev Comp Immunol (2014) 43:300–12. doi: 10.1016/j.dci.2013.08.019 24001580

[5] Barker SE Bricknell IR Covello J Purcell S Fast MD Wolters W. Sea Lice, Lepeophtheirus Salmonis (Kroyer 1837), Infected Atlantic Salmon (Salmo Salar L.) Are More Susceptible to Infectious Salmon Anemia Virus. PLoS One (2019) 14:e0209178. doi: 10.1371/journal.pone.0213232 30650077 PMC6334929

[6] Valenzuela-Miranda D Boltana S Cabrejos ME Yanez JM Gallardo-Escarate C. High-Throughput Transcriptome Analysis of ISAV-Infected Atlantic Salmon Salmo Salar Unravels Divergent Immune Responses Associated to Head-Kidney, Liver and Gills Tissues. Fish Shellfish Immunol (2015) 45:367–77. doi: 10.1016/j.fsi.2015.04.003 25910847

[7] Valdes-Donoso P Mardones FO Jarpa M Ulloa M Carpenter TE Perez AM. Co-Infection Patterns of Infectious Salmon Anaemia and Sea Lice in Farmed Atlantic Salmon, Salmo Salar L., in Southern Chil -2009). J Fish Dis (2013) 36:353–60. doi: 10.1111/jfd.12070 23347268

[8] Fast MD Johnson SC Eddy TD Pinto D Ross NW. Lepeophtheirus Salmonis Secretory/Excretory Products and Their Effects on Atlantic Salmon Immune Gene Regulation. Parasite Immunol (2007) 29:179–89. doi: 10.1111/j.1365-3024.2007.00932.x 17371455

[9] Peterson JW. “Bacterial Pathogenesis”. In: Medical Microbiology, 4th edition. University of Texas Medical Branch at Galveston (1996). 21413346

[10] Figueroa C Bustos P Torrealba D Dixon B Soto C Conejeros P. Coinfection Takes Its Toll: Sea Lice Override the Protective Effects of Vaccination Against a Bacterial Pathogen in Atlantic Salmon. Sci Rep (2017) 7:17817. doi: 10.1038/s41598-017-18180-6 29259257 PMC5736581

[11] Aaen SM Helgesen KO Bakke MJ Kaur K Horsberg TE. Drug Resistance in Sea Lice: A Threat to Salmonid Aquaculture. Trends Parasitol (2015) 31:72–81. doi: 10.1016/j.pt.2014.12.006 25639521

[12] Mcnair CM. Ectoparasites of Medical and Veterinary Importance: Drug Resistance and the Need for Alternative Control Methods. J Pharm Pharmacol (2015) 67:351–63. doi: 10.1111/jphp.12368 25644683

[13] Sutherland BJG Covello JM Friend SE Poley JD Koczka KW Purcell SL. Host–parasite Transcriptomics During Immunostimulant-Enhanced Rejection of Salmon Lice (Lepeophtheirus Salmonis) by Atlantic Salmon (Salmo Salar). FACETS (2017) 2:477–95. doi: 10.1139/facets-2017-0020

[14] Olmos Soto J Paniagua-Michel JDJ Lopez L Ochoa L. Functional Feeds in Aquaculture. In: Kim S-K, editor. Springer Handbook of Marine Biotechnology. Berlin, Heidelberg: Springer Berlin Heidelberg (2015). p. 1303–19.

[15] Caballero-Solares A Xue X Parrish CC Foroutani MB Taylor RG Rise ML. Changes in the Liver Transcriptome of Farmed Atlantic Salmon (Salmo Salar) Fed Experimental Diets Based on Terrestrial Alternatives to Fish Meal and Fish Oil. BMC Genomics (2018) 19:796. doi: 10.1186/s12864-018-5188-6 30390635 PMC6215684

[16] Katan T Xue X Caballero-Solares A Taylor RG Rise ML Parrish CC. Influence of Dietary Long-Chain Polyunsaturated Fatty Acids and ω6 to ω3 Ratios on Head Kidney Lipid Composition and Expression of Fatty Acid and Eicosanoid Metabolism Genes in Atlantic Salmon (Salmo Salar). Front Mol Biosci (2020) 7. doi: 10.3389/fmolb.2020.602587 PMC7767880 33381522

[17] Whyte SK Westcott JD Byrne P Hammell KL. Comparison of the Depletion of Emamectin Benzoate (SLICE®) Residues From Skeletal Muscle and Skin of Atlantic Salmon (Salmo Salar), for Multiple Dietary Dose Regimens at 10°C. Aquaculture (2011) 315:228–35. doi: 10.1016/j.aquaculture.2011.02.043

[18] Ritchie RJ Mcdonald JT Glebe B Young-Lai W Johnsen E Gagne N. Comparative Virulence of Infectious Salmon Anaemia Virus Isolates in Atlantic Salmon, Salmo Salar L. J Fish Dis (2009) 32:157–71. doi: 10.1111/j.1365-2761.2008.00973.x 19261043

[19] Hierholzer JC Killington RA. 2 - Virus Isolation and Quantitation. In: Mahy BWJ Kangro HO, editors. Virology Methods Manual. London: Academic Press (1996). p. 25–46.

[20] Leblanc F Arseneau JR Leadbeater S Glebe B Laflamme M Gagne N. Transcriptional Response of Atlantic Salmon (Salmo Salar) After Primary Versus Secondary Exposure to Infectious Salmon Anemia Virus (ISAV). Mol Immunol (2012) 51:197–209. doi: 10.1016/j.molimm.2012.03.021 22475434

[21] Bolger AM Lohse M Usadel B. Trimmomatic: A Flexible Trimmer for Illumina Sequence Data. Bioinformatics (2014) 30:2114–20. doi: 10.1093/bioinformatics/btu170 PMC4103590 24695404

[22] Trapnell C Pachter L Salzberg SL. TopHat: Discovering Splice Junctions With RNA-Seq. Bioinformatics (2009) 25:1105–11. doi: 10.1093/bioinformatics/btp120 PMC2672628 19289445

[23] Trapnell C Roberts A Goff L Pertea G Kim D Kelley DR. Differential Gene and Transcript Expression Analysis of RNA-Seq Experiments With TopHat and Cufflinks. Nat Protoc (2012) 7:562–78. doi: 10.1038/nprot.2012.016 PMC3334321 22383036

[24] Goff L Trapnell C Kelley D. CummeRbund: Visualization and Exploration of Cufflinks High-Throughput Sequencing Data. (2012). doi: 10.18129/B9.bioc.cummeRbund

[25] Yu G Wang LG Han Y He QY. Clusterprofiler: An R Package for Comparing Biological Themes Among Gene Clusters. OMICS (2012) 16:284–7. doi: 10.1089/omi.2011.0118 PMC3339379 22455463

[26] Morgan M Carlson M Tenenbaum D Arora S. AnnotationHub: Client to Access AnnotationHub Resources. R Package Version 2.4.2. (2016).

[27] Bindea G Mlecnik B Hackl H Charoentong P Tosolini M Kirilovsky A. ClueGO: A Cytoscape Plug-in to Decipher Functionally Grouped Gene Ontology and Pathway Annotation Networks. Bioinformatics (2009) 25:1091–3. doi: 10.1093/bioinformatics/btp101 PMC2666812 19237447

[28] Shannon P Markiel A Ozier O Baliga NS Wang JT Ramage D. Cytoscape: A Software Environment for Integrated Models of Biomolecular Interaction Networks. Genome Res (2003) 13:2498–504. doi: 10.1101/gr.1239303 PMC403769 14597658

[29] Untergasser A Cutcutache I Koressaar T Ye J Faircloth BC Remm M. Primer3–new Capabilities and Interfaces. Nucleic Acids Res (2012) 40:e115. doi: 10.1093/nar/gks596 22730293 PMC3424584

[30] Ye J Coulouris G Zaretskaya I Cutcutache I Rozen S Madden TL. Primer-BLAST: A Tool to Design Target-Specific Primers for Polymerase Chain Reaction. BMC Bioinf (2012) 13:134. doi: 10.1186/1471-2105-13-134 PMC3412702 22708584

[31] Pfaffl MW. A New Mathematical Model for Relative Quantification in Real-Time RT–PCR. Nucleic Acids Res (2001) 29:e45–5. doi: 10.1093/nar/29.9.e45 PMC55695 11328886

[32] Brown ABJ Whyte SK Braden LM Groman DB Purcell SL Fast MD. Vaccination Strategy Is an Important Determinant in Immunological Outcome and Survival in Arctic Charr (Salvelinus Alpinus) When Challenged With Atypical Aeromonas Salmonicida. Aquaculture (2020) 518:734838. doi: 10.1016/j.aquaculture.2019.734838

[33] Vandesompele J De Preter K Pattyn F Poppe B Van Roy N De Paepe A. Accurate Normalization of Real-Time Quantitative RT-PCR Data by Geometric Averaging of Multiple Internal Control Genes. Genome Biol (2002) 3:1–12. doi: 10.1186/gb-2002-3-7-research0034 PMC126239 12184808

[34] Carvalho LA Whyte SK Braden LM Purcell SL Taylor RG Rise ML. Submitted. Functional Feed Impacts on Atlantic Salmon (*Salmo Salar*) Systemic Immune Responses to Different Levels of Single Infection With Sea Lice (*Lepeophtheirus Salmonis*) and Co-Infection With Sea Lice and Infectious Salmon Anemia Virus. Fish Shellfish Immunol Rep.

[35] Gomez D Sunyer JO Salinas I. The Mucosal Immune System of Fish: The Evolution of Tolerating Commensals While Fighting Pathogens. Fish Shellfish Immunol (2013) 35:1729–39. doi: 10.1016/j.fsi.2013.09.032 PMC3963484 24099804

[36] Quiniou SMA Bigler S Clem LW Bly JE. Effects of Water Temperature on Mucous Cell Distribution in Channel Catfish Epidermis: A Factor in Winter Saprolegniasis. Fish Shellfish Immunol (1998) 8:1–11. doi: 10.1006/fsim.1997.0115

[37] Iger Y Wendelaar Bonga SE. Cellular Responses of the Skin of Carp (Cyprinus Carpio) Exposed to Acidified Water. Cell Tissue Res (1994) 275:481–92. doi: 10.1007/BF00318817

[38] Landeira-Dabarca A Alvarez M Molist P. Food Deprivation Causes Rapid Changes in the Abundance and Glucidic Composition of the Cutaneous Mucous Cells of Atlantic Salmon Salmo Salar L. J Fish Dis (2014) 37:899–909. doi: 10.1111/jfd.12184 24117614

[39] Liu L Li C Su B Beck BH Peatman E. Short-Term Feed Deprivation Alters Immune Status of Surface Mucosa in Channel Catfish (Ictalurus Punctatus). PLoS One (2013) 8:e74581. doi: 10.1371/journal.pone.0074581 24023952 PMC3762756

[40] Jodaa Holm H Wadsworth S Bjelland AK Krasnov A Evensen O Skugor S. Dietary Phytochemicals Modulate Skin Gene Expression Profiles and Result in Reduced Lice Counts After Experimental Infection in Atlantic Salmon. Parasit Vectors (2016) 9:271. doi: 10.1186/s13071-016-1537-y 27164990 PMC4862074

[41] Covello J Friend S Purcell S Burka J Markham R Donkin A. Effects of Orally Administered Immunostimulants on Inflammatory Gene Expression and Sea Lice (Lepeophtheirus Salmonis) Burdens on Atlantic Salmon (Salmo Salar). Aquaculture (2012) 366:9–16. doi: 10.1016/j.aquaculture.2012.08.051

[42] Chen F Lu XJ Nie L Ning YJ Chen J. Molecular Characterization of a CC Motif Chemokine 19-Like Gene in Ayu (Plecoglossus Altivelis) and Its Role in Leukocyte Trafficking. Fish Shellfish Immunol (2018) 72:301–8. doi: 10.1016/j.fsi.2017.11.012 29128493

[43] Schutyser E Struyf S Van Damme J. The CC Chemokine CCL20 and Its Receptor CCR6. Cytokine Growth Factor Rev (2003) 14:409–26. doi: 10.1016/S1359-6101(03)00049-2 12948524

[44] Hsu YJ Hou CY Lin SJ Kuo WC Lin HT Lin JH. The Biofunction of Orange-Spotted Grouper (Epinephelus Coioides) CC Chemokine Ligand 4 (CCL4) in Innate and Adaptive Immunity. Fish Shellfish Immunol (2013) 35:1891–8. doi: 10.1016/j.fsi.2013.09.020 24120504

[45] Krasnov A Skugor S Todorcevic M Glover KA Nilsen F. Gene Expression in Atlantic Salmon Skin in Response to Infection With the Parasitic Copepod Lepeophtheirus Salmonis, Cortisol Implant, and Their Combination. BMC Genomics (2012) 13:130. doi: 10.1186/1471-2164-13-130 22480234 PMC3338085

[46] Wagner GN Fast MD Johnson SC. Physiology and Immunology of Lepeophtheirus Salmonis Infections of Salmonids. Trends Parasitol (2008) 24:176–83. doi: 10.1016/j.pt.2007.12.010 18329341

[47] Cutroneo KR Rokowski R Counts DF. Glucocorticoids and Collagen Synthesis: Comparison of In Vivo and Cell Culture Studies. Coll Relat Res (1981) 1:557–68. doi: 10.1016/S0174-173X(81)80037-4 7049552

[48] Sveen LR Timmerhaus G Krasnov A Takle H Handeland S Ytteborg E. Wound Healing in Post-Smolt Atlantic Salmon (Salmo Salar L.). Sci Rep (2019) 9:3565. doi: 10.1038/s41598-019-39080-x 30837496 PMC6400935

[49] Gillies AR Lieber RL. Structure and Function of the Skeletal Muscle Extracellular Matrix. Muscle Nerve (2011) 44:318–31. doi: 10.1002/mus.22094 PMC3177172 21949456

[50] Manske RC. Postsurgical Orthopedic Sports Rehabilitation: Knee & Shoulder. St. Louis, Missouri: Mosby (2006).

[51] Holm H Santi N Kjoglum S Perisic N Skugor S Evensen O. Difference in Skin Immune Responses to Infection With Salmon Louse (Lepeophtheirus Salmonis) in Atlantic Salmon (Salmo Salar L.) of Families Selected for Resistance and Susceptibility. Fish Shellfish Immunol (2015) 42:384–94. doi: 10.1016/j.fsi.2014.10.038 25449368

[52] Robledo D Gutierrez AP Barria A Yanez JM Houston RD. Gene Expression Response to Sea Lice in Atlantic Salmon Skin: RNA Sequencing Comparison Between Resistant and Susceptible Animals. Front Genet (2018) 9:287. doi: 10.3389/fgene.2018.00287 30123239 PMC6086009

[53] Umasuthan N Xue X Caballero-Solares A Kumar S Westcott JD Chen Z. Transcriptomic Profiling in Fins of Atlantic Salmon Parasitized With Sea Lice: Evidence for an Early Imbalance Between Chalimus-Induced Immunomodulation and the Host's Defense Response. Int J Mol Sci (2020) 21:2417–2462. doi: 10.3390/ijms21072417 PMC7177938 32244468

[54] Peatman E Lange M Zhao H Beck BH. Physiology and Immunology of Mucosal Barriers in Catfish (Ictalurus Spp.). Tissue Barriers (2015) 3:e1068907. doi: 10.1080/21688370.2015.1068907 26716071 PMC4681283

[55] Haller O Kochs G Weber F. The Interferon Response Circuit: Induction and Suppression by Pathogenic Viruses. Virology (2006) 344:119–30. doi: 10.1016/j.virol.2005.09.024 PMC7125643 16364743

[56] Kileng O Brundtland MI Robertsen B. Infectious Salmon Anemia Virus Is a Powerful Inducer of Key Genes of the Type I Interferon System of Atlantic Salmon, But Is Not Inhibited by Interferon. Fish Shellfish Immunol (2007) 23:378–89. doi: 10.1016/j.fsi.2006.11.011 17257858

[57] Gack MU Albrecht RA Urano T Inn KS Huang IC Carnero E. Influenza A Virus NS1 Targets the Ubiquitin Ligase TRIM25 to Evade Recognition by the Host Viral RNA Sensor RIG-I. Cell Host Microbe (2009) 5:439–49. doi: 10.1016/j.chom.2009.04.006 PMC2737813 19454348

[58] Van Der Aa LM Levraud JP Yahmi M Lauret E Briolat V Herbomel P. A Large New Subset of TRIM Genes Highly Diversified by Duplication and Positive Selection in Teleost Fish. BMC Biol (2009) 7:7. doi: 10.1186/1741-7007-7-7 19196451 PMC2657112

[59] Yang Y Huang Y Yu Y Yang M Zhou S Qin Q. RING Domain Is Essential for the Antiviral Activity of TRIM25 From Orange Spotted Grouper. Fish Shellfish Immunol (2016) 55:304–14. doi: 10.1016/j.fsi.2016.06.005 27276113

[60] Huang Y Zhang J Liu J Hu Y Ni S Yang Y. Fish TRIM35 Negatively Regulates the Interferon Signaling Pathway in Response to Grouper Nodavirus Infection. Fish Shellfish Immunol (2017) 69:142–52. doi: 10.1016/j.fsi.2017.08.019 28823982

[61] Gack MU Shin YC Joo CH Urano T Liang C Sun L. TRIM25 RING-Finger E3 Ubiquitin Ligase Is Essential for RIG-I-Mediated Antiviral Activity. Nature (2007) 446:916–20. doi: 10.1038/nature05732 17392790

[62] Krasnov A Timmerhaus G Schiotz BL Torgersen J Afanasyev S Iliev D. Genomic Survey of Early Responses to Viruses in Atlantic Salmon, Salmo Salar L. Mol Immunol (2011) 49:163–74. doi: 10.1016/j.molimm.2011.08.007 21924497

[63] Aoki T Takano T Hikima J-I. DNA Vaccine-Mediated Innate Immune Response Triggered by PRRs in Teleosts. Fisheries Sci (2015) 81:205–17. doi: 10.1007/s12562-014-0845-4

[64] Dahle MK Wessel O Timmerhaus G Nyman IB Jorgensen SM Rimstad E. Transcriptome Analyses of Atlantic Salmon (Salmo Salar L.) Erythrocytes Infected With Piscine Orthoreovirus (PRV). Fish Shellfish Immunol (2015) 45:780–90. doi: 10.1016/j.fsi.2015.05.049 26057463

[65] Chen SN Zou PF Nie P. Retinoic Acid-Inducible Gene I (RIG-I)-Like Receptors (RLRs) in Fish: Current Knowledge and Future Perspectives. Immunology (2017) 151:16–25. doi: 10.1111/imm.12714 28109007 PMC5382327

[66] Hoffpauir CT Bell SL West KO Jing T Wagner AR Torres-Odio S. TRIM14 Is a Key Regulator of the Type I IFN Response During Mycobacterium Tuberculosis Infection. J Immunol (2020) 205:153–67. doi: 10.4049/jimmunol.1901511 PMC7313415 32404352

[67] Leblanc F Laflamme M Gagne N. Genetic Markers of the Immune Response of Atlantic Salmon (Salmo Salar) to Infectious Salmon Anemia Virus (ISAV). Fish Shellfish Immunol (2010) 29:217–32. doi: 10.1016/j.fsi.2010.03.007 20371292

[68] Lauscher A Krossoy B Frost P Grove S Konig M Bohlin J. Immune Responses in Atlantic Salmon (Salmo Salar) Following Protective Vaccination Against Infectious Salmon Anemia (ISA) and Subsequent ISA Virus Infection. Vaccine (2011) 29:6392–401. doi: 10.1016/j.vaccine.2011.04.074 21554914

[69] Lhorente JP Gallardo JA Villanueva B Carabano MJ Neira R. Disease Resistance in Atlantic Salmon (Salmo Salar): Coinfection of the Intracellular Bacterial Pathogen Piscirickettsia Salmonis and the Sea Louse Caligus Rogercresseyi. PLoS One (2014) 9:e95397. doi: 10.1371/journal.pone.0095397 24736323 PMC3988197

