# Supplementary material for: Transcriptome Analysis of Atlantic Salmon (Salmo salar) Skin in Response to Sea Lice and Infectious Salmon Anemia Virus Co-Infection Under Different Experimental Functional Diets
Source: Front Immunol. 2022 Jan 3;12:787033. doi: 10.3389/fimmu.2021.787033 (PMC8763012; doi:10.3389/fimmu.2021.787033)
Supplement: Supplementary file 1 [file DataSheet_1.zip › Supplementary Files/S_table.docx]

Supplementary table 1. Primers used for qPCR validation (5’ to 3’).

| Gene  abbreviation | Gene name | Forward | Reserve |
| --- | --- | --- | --- |
| *fgfbp1* | Fibroblast growth factor-binding protein 1 precursor | ATGGTGGCTCACTGTCAAGG | CACATTTCCTTTGGGGCTGC |
| *c4* | complement C4 | GACGGACGAGACCGAAGAGA | TTAAACCTGGCCCCATCGTG |
| *c6* | complement component C6 precursor | AGTGCGATGCTGTTCTGACA | ATACAGCGACCATTGCCACA |
| *mbl2* | mannose-binding protein C | TTGAAAGGCCATCCCCTCAC | GGGCATTGAAGGAGCACTCA |
| *ccr6* | C-C chemokine receptor type 6 | TTCAGGTTCCCAGACAACGC | GCCTCTGGAAGTTCTTGGCA |
| *cd5* | T-cell surface glycoprotein CD5 | TACCTCCGGTCCCATCACTC | GTCCCCCTTACAGGACTTTCC |
| *ccr9* | C-C chemokine receptor type 9 | GGCTTACACACACGCCTGC | TCCCTGGATCTTGCTGAGTTTC |
| *tagap* | T-cell activation Rho GTPase-activating protein | TCCTACAGGTGGACAGCAGA | TTGCGTTCATAGAGGCTGGG |
| *ccl4* | C-C motif chemokine 4 | GTGGATCGGTAACCTCCTGC | GCAGATTCGTTTGCCCTTCA |
| *eif** | Eukaryotic translation initiation factor 3 subunit 6 | GTCGCCGTACCAGCAGGTGATT | CGTGGGCCATCTTCTTCTCGA |
| *rps20** | Ribosomal protein s20 | GCAGACCTTATCCGTGGAGCTA | TGGTGATGCGCAGAGTCTTG |

*indicates reference genes
