# Supplementary material for: Transcriptome Analysis of Atlantic Salmon (Salmo salar) Skin in Response to Sea Lice and Infectious Salmon Anemia Virus Co-Infection Under Different Experimental Functional Diets
Source: Front Immunol. 2022 Jan 3;12:787033. doi: 10.3389/fimmu.2021.787033 (PMC8763012; doi:10.3389/fimmu.2021.787033)
Supplement: Supplementary file 1 [file DataSheet_1.zip › Supplementary Files/Supp_Fig_legend.docx]

**Supplementary Figures legends**

Supplementary Figure S1. Principal component analysis (PCA) of the skin samples from fish receiving experimental diets for 28 days before infection.

Supplementary Figure 2. Hierarchical clustering of diet-specific genes in pre-infected samples visualized as a heatmap. The columns and rows depict the different diet group and differentially expressed genes (DEGs) expression values (FPKM). The heatmap colors red and blue indicate up-regulated and down-regulated genes espectively.

Supplementary Figure 3. A high resolution version of hierarchical clustering of shared differentially expressed genes (DEGs) identified in three groups (single vs. pre-infection, co-infection vs. pre-infection, and co-infection vs. single infection).
