# Supplementary material for: Transcriptome Analysis of Atlantic Salmon (Salmo salar) Skin in Response to Sea Lice and Infectious Salmon Anemia Virus Co-Infection Under Different Experimental Functional Diets
Source: Front Immunol. 2022 Jan 3;12:787033. doi: 10.3389/fimmu.2021.787033 (PMC8763012; doi:10.3389/fimmu.2021.787033)

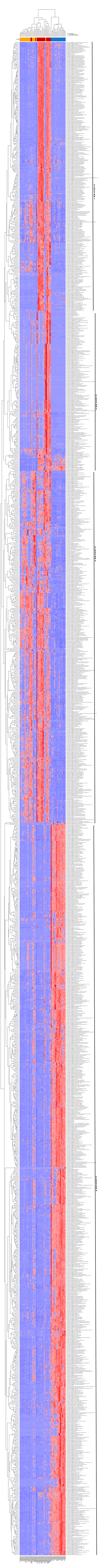

Supplement: Supplementary file 1 [file Figure S3.PDF › Supplementary Files/Figure S3.PDF]
